# Supplementary material for: Studies on Reproductive Development and Breeding Habit of the Commercially Important Bamboo Bambusa tulda Roxb
Source: Plants (Basel). 2021 Nov 4;10(11):2375. doi: 10.3390/plants10112375 (PMC8619091; doi:10.3390/plants10112375)
Supplement: Supplementary file 1 [file plants-10-02375-s001.zip › plants-1330880-supplementary materials Table S1.pdf]

**Table S1.** Percentage of pollen germination of *Bambusa tulda* in Brewbaker and Kwack's medium supplemented with 10, 15, 20, 25 and 30% sucrose (w/v).

| Population | Percentage (%) of sucrose in Brewbaker and Kwack's medium | | | | | | | | | |
|---|---|---|---|---|---|---|---|---|---|---|
| | 10 | | 15 | | 20 | | 25 | | 30 | |
| | Total number of pollens observed | Total number of germinated pollens | Total number of pollens observed | Total number of germinated pollens | Total number of pollens observed | Total number of germinated pollens | Total number of pollens observed | Total number of germinated pollens | Total number of pollens observed | Total number of germinated pollens |
| SHYM7 | 119 | 16 | 112 | 16 | 74 | 9 | 109 | 9 | 86 | 3 |
| SHYM16 | 87 | 11 | 71 | 11 | 66 | 7 | 59 | 4 | 86 | 2 |
| BNDL24 | 76 | 10 | 57 | 9 | 63 | 8 | 46 | 4 | 64 | 2 |
| Percentage (%) of pollen germination | 12.64±0.41 | | 14.29±0.80 | | 10.61±1.09 | | 6.78±1 | | 2.33±0.59 | |
