# Supplementary material for: Studies on Reproductive Development and Breeding Habit of the Commercially Important Bamboo Bambusa tulda Roxb
Source: Plants (Basel). 2021 Nov 4;10(11):2375. doi: 10.3390/plants10112375 (PMC8619091; doi:10.3390/plants10112375)
Supplement: Supplementary file 1 [file plants-10-02375-s001.zip › plants-1330880-supplementary materials Table S2.pdf]

Table S2. Various flowering events recorded in *Bambusa tulda* in India

Place	Location	Year	Gregarious/ Sporadic	Reference
Bengal	-	1867-1868	Gregarious	Troup, 1921
Bengal	-	1872	Gregarious	Troup, 1921
Mizoram	-	1880	Gregarious	Mohan Ram and Gopal, 1981
Bengal	-	1884	Gregarious	Troup, 1921
Mizoram	-	1884	Gregarious	Mohan Ram and Gopal, 1981
Assam	-	1889	Gregarious	Troup, 1921
Mizoram	-	1924-1928	Gregarious	Perry, 1931
Mizoram	-	1928	Gregarious	Mohan Ram and Gopal, 1981
Mizoram	-	1976-1979	Gregarious	Mohan Ram and Gopal, 1981
Arunachal Pradesh	Doimuk, Itanagar	1986	Gregarious	Naithani, 1993
Uttarakhand	Dehradun	1987	Gregarious	Rawat, 1987
Assam	North Cachar hills	1987	-	Gupta, 1987
Assam	Bajali area	2008	Gregarious	Sarma *et al.*, 2010
Arunachal Pradesh	Lower and Upper Dibang Valley	2011-2012	Gregarious	Naithani *et al.*, 2013
Mizoram	-	1880	Gregarious	Mohan Ram and Gopal, 1981

West Bengal	Dighra, Hooghly	2003	Sporadic	Bhattacharya *et al*., 2006
West Bengal	Rahuta, Shyamnagar	2013-16	Sporadic	This study
West Bengal	Paltapara, Shyamnagar	2013	Sporadic	This study
West Bengal	Rajhat, Bandel	2010-19	Sporadic	This study
West Bengal	Kumra Kashipur, Habra	2015	Sporadic	This study
West Bengal	Rahuta, Shyamnagar	2016-19	Sporadic	This study
West Bengal	Rajhat, Bandel	2017- till to date	Sporadic	This study
West Bengal	Rahuta, Shyamnagar	2018- till to date	Sporadic	This study
West Bengal	Rahuta, Shyamnagar	2021	Sporadic	This study
West Bengal	Rahuta, Shyamnagar	2021	Sporadic	This study
