# Supplementary figures and images for: Studies on Reproductive Development and Breeding Habit of the Commercially Important Bamboo Bambusa tulda Roxb
Source: Plants (Basel). 2021 Nov 4;10(11):2375. doi: 10.3390/plants10112375 (PMC8619091; doi:10.3390/plants10112375)

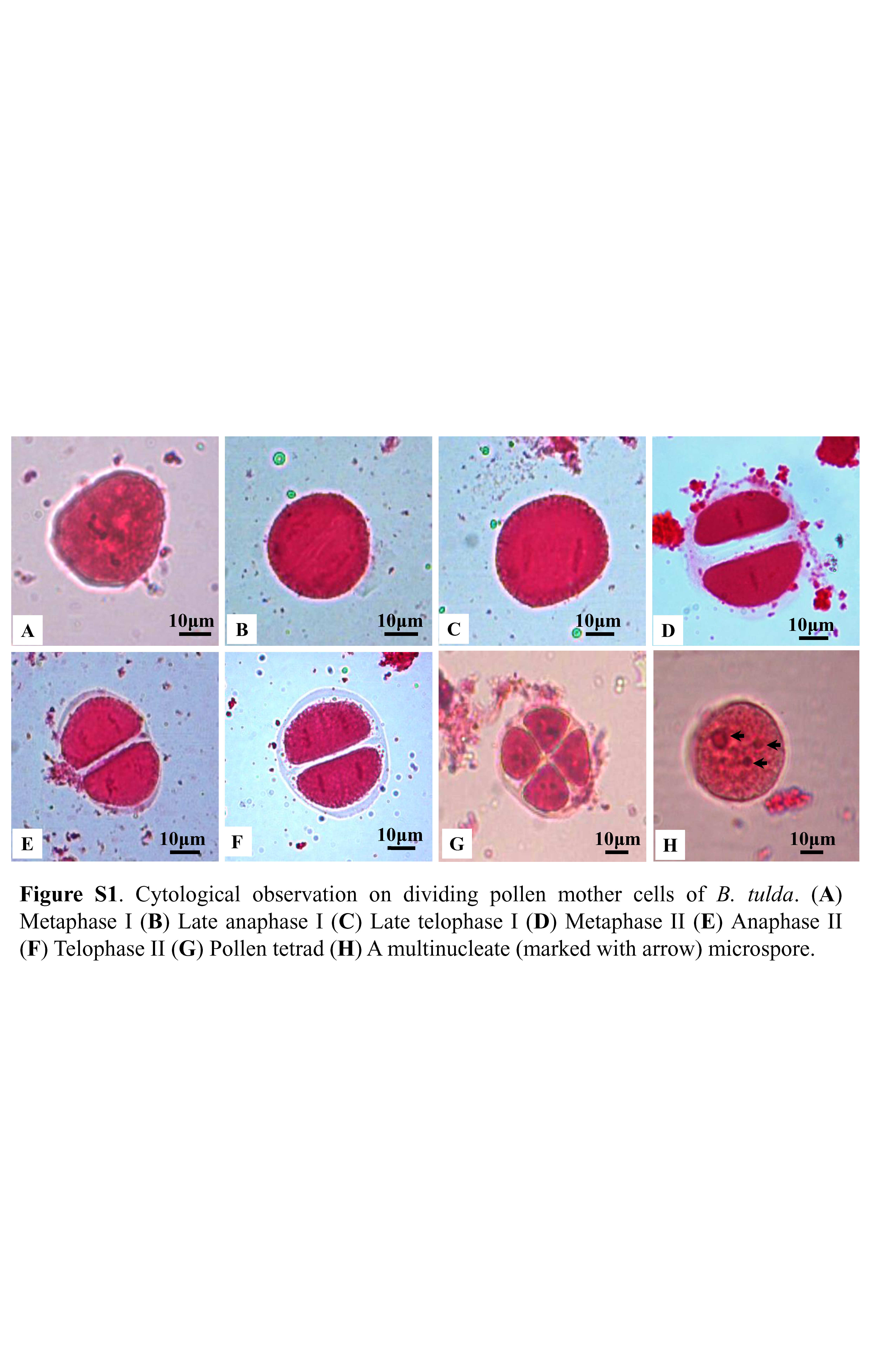

Supplement: Supplementary file 1 [file plants-10-02375-s001.zip › Chakraborty et al._FIG. S1_R1.tif]
